# Predictors of Medical Students’ Perceptions About Medical Cannabis

**DOI:** 10.7759/cureus.24390

**Published:** 2022-04-22

**Authors:** Robin J Jacobs, Michael N Kane, Joshua Caballero

**Affiliations:** 1 Medical and Behavioral Research, Health Informatics, Medical Education, Nova Southeastern University, Fort Lauderdale, USA; 2 Social Work, Florida Atlantic University, Boca Raton, USA; 3 Pharmacy, University of Georgia, Athens, USA

**Keywords:** pain management, public health, training, knowledge, beliefs, attitudes, medical education, medical student, marijuana, cannabis

## Abstract

Background: There has been a recent uptick in interest regarding the therapeutic properties of cannabis. Evidence exists to support the role of medical cannabis (MC) in chronic illness management for conditions such as posttraumatic stress, pain, and cancer. The majority of physicians in the United States report not knowing how to prescribe or answer questions about MC and receive minimal education about it during training. As MC becomes more socially acceptable with federal legalization in process, new physicians will encounter patients looking for information on the utility and safety of MC. The goal of this research was thus to assess the perceived knowledge, beliefs, and perceptions of medical students towards MC, and to obtain a better understanding of factors that may influence their attitudes.

Methods: A descriptive, cross-sectional study design was used to investigate the medical students’ knowledge, attitudes, and perceptions regarding MC. Quantitative data were collected from 526 medical students (years one to four) via an anonymous, online, 32-item questionnaire to determine if perceived knowledge, concerns about the potential negative effects of cannabis, and certain beliefs would significantly contribute to their attitudes toward MC. Hypothesis testing was conducted using Spearman-rank order correlation and multivariate linear regression analyses.

Results: A statistically significant regression equation was found: (*F*(4, 428)=114.826, *p*<.001 with an *R^2^*=0.518 [adjusted *R^2 ^*=0.513]) indicating greater perception of knowledge about MC, lower concern for possible negative effects of MC use, greater belief in federal legalization of MC, and greater belief in the federal legalization of recreational cannabis significantly contributed to a higher score on positive attitudes and perceptions toward MC. Moreover, while many participants reported physicians should be able to prescribe MC, they reported that little if any MC education had been provided.

Conclusions: This study identified the knowledge, concerns, and perceptions of medical students regarding MC as well as several factors contributing to their attitudes about it. Favorable attitudes toward MC among patients exist and as its popularity and acceptance among patients continue, more may be asking their physicians about symptomatic and curative treatment with cannabis-based products. Results from this research have the potential to assist medical educators in understanding students’ perceptions about MC to help guide innovative and contemporary curricular advances as a public health imperative.

## Introduction

Cannabis is a substance that has been used for both recreational use and medicinal purposes. Published research studies have indicated that cannabis can be used to alleviate pain and manage certain chronic health conditions and its benefits may outweigh its potential harms [1-7]. Currently, legal, qualified physicians may recommend medical cannabis (MC) for patients provided they have a diagnosed qualifying condition such as multiple sclerosis, post-traumatic stress disorder, amyotrophic lateral sclerosis, and Parkinson’s disease. Additionally, a patient with a terminal condition and chronic (nonmalignant) pain resulting from a qualifying medical condition is also included [8]. Qualified allopathic or osteopathic physicians must comply with all physician education requirements (i.e., additional training in MC) to be certified to recommend MC. As of February 2022, 39 states plus Washington, D.C., have legalized MC [9]. At this time, MC is federally classified as a Schedule I substance. However, in April 2022, the House of Representatives passed legislation (220-204 vote) that would legalize marijuana nationwide [10].

Published research exists depicting favorable attitudes toward MC among patients and as its popularity and acceptance among patients continue, more may be asking their physicians about symptomatic and curative treatment with cannabis-based products [11-19]. Despite the increasing acceptance of MC and the relaxation of restrictions on its use nationwide, the medical community at large has yet to reach a consensus on the appropriate medical uses of marijuana. However, published reports indicate that medical students hold favorable attitudes toward the benefits of MC, but do not receive formal training in MC, do not feel equipped to discuss MC with patients, and want training regarding MC in medical school. Medical students in general are more likely to report elevated confidence in knowledge about MC and in cannabis’ medical efficacy as compared to physicians [20].

As MC becomes more socially acceptable [21], new physicians will encounter a growing number of patients looking for information on its utility and safety. [22]. Moreover, medical students tend to be young, and young adults may hold more permissive opinions about cannabis [23] and perceive it to be less harmful than their older counterparts [24]. It is thus important to assess medical students’ perceived knowledge and perceptions about MC, including their opinion about federal legislation and regulation, if medical educators are to adequately prepare students to use it in their future practice [25].

Given these factors, MC seems to be a critical issue for the medical profession and particularly medical students who, after completing their training, might be expected to prescribe MC and manage therapies. However, there is limited published research on the knowledge and perceptions of United States medical students about MC, including their willingness to use MC in future practice. Moreover, the existing research on medical students’ perceptions of MC is limited to descriptive data or, in some cases, bivariate, non-predictive relationships [25-30].

In a recent systematic review exploring medical professionals and students’ perceptions and knowledge about MC (worldwide), more than half were conducted exclusively in the US and only two of those were conducted with medical students, one of which was a dissertation study comparing a small sample of medical students to social work students regarding attitudes toward MC [20,29]. Other studies focused on students’ attitudes about MC and specific diseases [18], or pain management only [26,28]. No study to our knowledge has used multivariate modeling to predict medical students’ perceptions toward MC such as the one completed in this study.

Research questions

The aim of this cross-sectional, observational study was to investigate medical students’ perceived knowledge, concerns, and attitudes about MC. Specifically, this study sought to answer the questions: 1) What are the perceived knowledge and perceptions of medical students regarding medical cannabis? 2) Will knowledge of MC, concern for possible negative effects, and opinions about the legalization of cannabis make statistically significant contributions to perceptions about MC in medical students?

Hypothesis

It was hypothesized a priori that: (a) there would be statistically significant relationships between medical students’ perceived knowledge of MC, concern for possible negative effects of MC use, belief in federal legalization of MC, and belief in the federal legalization of recreational cannabis (the independent variables), and perceptions about medical cannabis (the dependent variable); (b) the above independent variables will make statistically significant contributions to medical students’ perceptions about MC.

## Materials and methods

Data were collected from medical students enrolled in a college of osteopathic medicine via an electronic link. This study was approved by the Nova Southeastern University Institutional Review Board (protocol number 2022-28).

Sample and questionnaire administration

Between January 25 and March 11, 2022, an anonymous questionnaire was administered via email to all the enrolled 1,447 medical students years one through four, using REDCap, a web application for creating and managing online surveys [31]. The application REDCap is a means for acquiring copious amounts of quantitative data from large populations. Participants provided informed consent for participation by agreeing to open the link to the survey. A cover letter was included in the email explaining the goal of the study and that their participation was strictly voluntary. The survey took about approximately five to 10 minutes to fill out. Reminder emails were given at predetermined intervals to promote student participation in completing questionnaires to help mitigate a low response rate.

Assessment instrument

Relevant published studies guided the development of the 32-item questionnaire (developed by the researchers) to assess students’ perceived knowledge and perceptions toward MC. The questionnaire used Likert-type items with a 6-point response set (1=strongly agree, 2=agree, 3=somewhat agree, 4=somewhat disagree, 5=disagree, 6=strongly disagree), categorical items (for which set choices were provided), and dichotomous items (e.g., yes/no), many of which were adapted from various published studies [13,30,32-56]. The following content areas were evaluated.

*Perceived Knowledge of MC* 

To assess the perceived knowledge of medical students, four items were included that ask students to report confidence in their knowledge of MC: 1) I am familiar with the possible therapeutic effects of MC; 2) I have substantial knowledge about MC; 3) I am extremely confident regarding my current knowledge of MC; 4) I have good knowledge of the side effects of medicinal marijuana. 

Concern About Possible Negative Effects of MC

The five items used to assess participants' concern for possible negative effects of MC use were: 1) Using cannabis poses serious mental health risks; 2) Using cannabis poses serious physical health risks; 3) MC use can be addictive; 4) I am concerned with MC’s potential for abuse/misuse; 5) I am concerned about the potential side effects of medical marijuana use.

Perceptions About MC 

Nine items assessed general attitudes and perceptions about MC among medical students: 1) Marijuana has an acceptable role in medicine: 2) MC helps patients who suffer from chronic, debilitating medical conditions; 3) There are significant physical health benefits using MC; 4) Training about MC should be incorporated into medical/health/social wellbeing related academic (preclinical) curricula; 5) Training about MC should be incorporated into residency/field practice (clinical) requirements; 6) Marijuana should be reclassified so that it is no longer a Schedule 1 drug (participants were provided an explanation of Schedule 1 drug); 7) Physicians should be able to legally prescribe marijuana as medical therapy; 8) Physicians should recommend medical marijuana as medical therapy; and 9) As a healthcare provider (in the future), I would be willing to help patients access MC.

Participants were also asked if they believed MC is effective for certain medical conditions, for which there was a list of 20 conditions displayed. Participants had the option to choose more than one health condition, with an option of “other.” Examples of the conditions to choose from were arthritis, cancer, chronic pain, fibromyalgia, glaucoma, and mental health conditions. In addition, items to collect data on participants’ personal characteristics (i.e., age, sex, race, ethnicity, year of study, personal use of or knowledge of someone who has used MC, and items about if medical and recreational marijuana should be made legal nationally, were included.

Preliminary analysis

Out of the 1,447 students enrolled in the program, 637 students returned the questionnaire (44% response rate). Of those, there were 111 cases with less than 66% completed items and were omitted, leaving a total of 526 completed questionnaires for the analysis (36% completion rate).

The Statistical Package for the Social Sciences (SPSS) version 20.0 (IBM Corp., Armonk, NY) [57] was utilized to analyze the data after extracting it from REDCap [31]. Visual inspections of the observed distributions and tests for skewness and kurtosis i.e., assessment for normal distributions, were employed. Reliability estimates (Cronbach α) for all scales in the survey were computed: the perceptions about the use of the MC scale (PU-S; α = .91); perceived knowledge of the MC scale (PK-S; α = .89); and concern for possible negative effects of the MC scale (CNE-S; α = .89). All reliability estimates were within acceptable limits (α >.70) [57].

Prior to performing the regression, multicollinearity testing was conducted. The independent variables were within acceptable variance inflation factors limits. Moreover, the scales i.e., PU-S, PK-S, CNE-S, were normally distributed, implying the data did not deviate from normality enough to affect inference.

Data analysis

Sample characteristics are reported as frequency and percentage (for discrete variables) and as means and standard deviation (for continuous variables). The results are presented as numbers and percentages, mean and standard deviation. Point-biserial correlation and multivariate linear regression were used for hypothesis testing. 

## Results

Descriptive statistics

Sample Demographics

The mean age of the participants was 26 years (range 18 to 47 years); 239 (45.4%) were women, 229 (43.5%) were men, and 58 (11%) preferred not to answer. Seventy-nine (n=15%) identified as Hispanic/Latinx; 330 (62.7%) identified as Caucasian, 96 (18.3) identified as Asian or Pacific Islander, 12 (2.3%) identified as Black, and 88 (16.7%) declined to report their race. Regarding academic level in the medical program, the breakdown is as follows: first year (n=169; 32.1%), second year (n=251; 47.7%), third year (n=73; 13.9%), and fourth year (n=33; 6.3%).

Efficacy of MC

**Figure 1:**
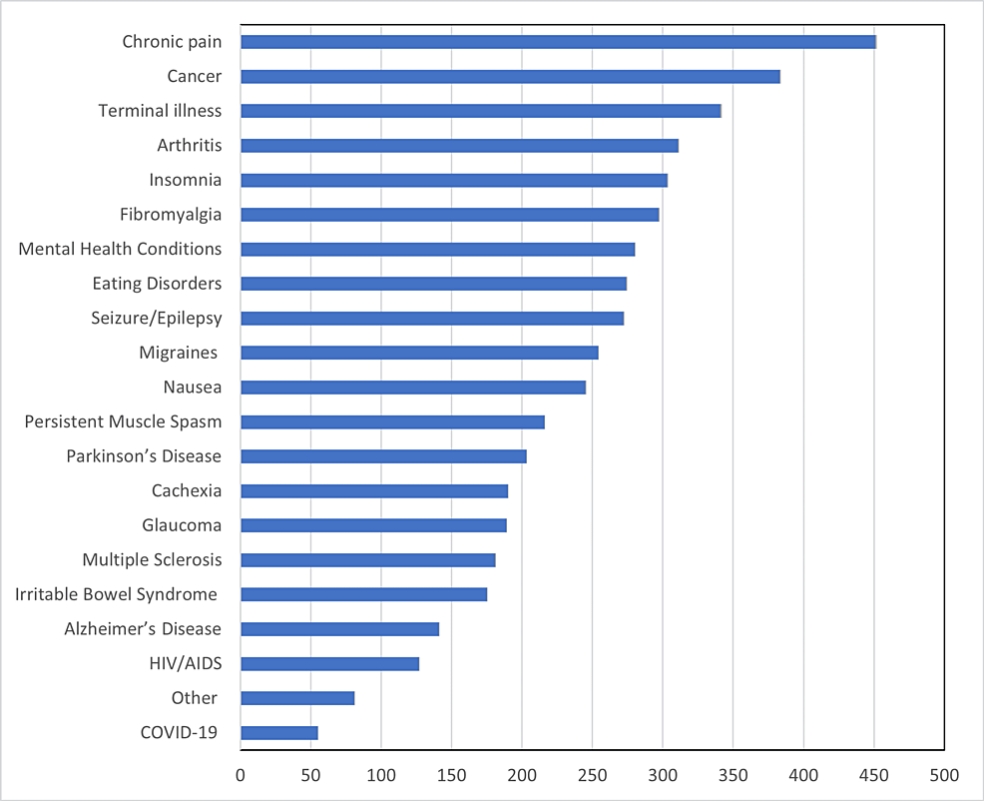
Responses to the item “Medical cannabis is effective for the following medical conditions (check all that apply)” (N= 501)

Regarding the efficacy of MC use with certain conditions, 90% percent of participants cited chronic pain (n=451); 76.4% (n=383) cited cancer, 68.1% (n=341) cited terminal illness, 62.1% (n=311) cited arthritis, 60.5% (n=303) cited insomnia, 59.3% (n=297) cited fibromyalgia; 55.9% (n=280) cited mental health conditions, 54.3% (n=n=272) cited seizures/epilepsy, and 50.7% (n=254) cited migraine. Less than 50% of participants reported any of the remaining conditions, although all conditions were selected (Figure 1).

Major study variables

Summary Statistics for Single Items 

Table 1 reports the summary statistics for major study items grouped by content area.

**Table 1:** Summary statistics for major study items grouped by content area Response set 1=strongly agree, 2=agree, 3=somewhat agree, 4=somewhat disagree, 5=disagree, 6=strongly disagree

Content Area and Item	n	Mean	SD	Min.	Max
Perceptions about MC Use Scale (PU-S) Items					
Marijuana has an acceptable role in medicine.	526	2.13	0.972	1	6
Medical marijuana helps patients who suffer from chronic, debilitating medical conditions.	526	1.84	0.863	1	6
There are significant physical health benefits to using medical cannabis	526	2.36	1.071	1	6
Training about medical marijuana should be incorporated into medical/health/social wellbeing-related academic (preclinical) curricula.	496	1.96	0.932	1	6
Training about medical marijuana should be incorporated into residency/field practice (clinical) requirements.	496	2.07	1.001	1	6
Marijuana should be reclassified so that it is no longer a Schedule 1 drug.	487	1.87	1.174	1	6
Physicians should be able to legally prescribe marijuana as medical therapy.	496	1.98	0.926	1	6
Physicians should recommend medical marijuana as medical therapy.	496	2.39	1.003	1	6
As a healthcare provider (in the future), I would be willing to help patients access medical marijuana.	496	2.22	1.052	1	6
Perceived Knowledge Scale (PK-S) Items					
I am familiar with the possible therapeutic effects of medical marijuana.	400	2.15	0.933	1	6
I have substantial knowledge about medical marijuana.	525	3.39	1.353	1	6
I am extremely confident regarding my current knowledge of medical marijuana.	526	3.70	1.427	1	6
I have good knowledge of the side effects of medicinal marijuana.	526	3.17	1.267	1	6
Concern for Negative Effects Scale (CNE-S) Items					
Using cannabis poses serious mental health risks.	526	3.68	1.271	1	6
Using cannabis poses serious physical health risks.	526	4.09	1.223	1	6
Medical marijuana use can be addictive.	487	2.87	1.184	1	6
I am concerned with medical marijuana’s potential for abuse/misuse.	487	2.99	1.387	1	6
I am concerned about the potential side effects of medical marijuana use.	526	3.15	1.388	1	6

Measures of Variability

Table 2 reports the summary statistics (i.e., mean, standard deviation) for the major study variables. The scores ranged from 1 to 6. For the PK-S and the attitudes and perceptions scale (AP-S), higher scores indicate greater perceived knowledge and more positive perceptions about MC. For the CNE-S, lower scores indicate greater concern. The mean score for the PK-S was 3.17 (SD=.526). The mean score for the CNE-S was 3.39 (SD=.526). Participants scored low overall on the (PU-S), resulting in a mean score of 2.1 (SD=.797).

**Table 2:** Summary statistics for the major study variables (N=526) Mean (SD)a: Represents the mean score for any item on a scale. For the perceived knowledge scale and the attitudes and perceptions scale (AP-S), higher scores indicate greater perceived knowledge and more positive attitudes and perceptions about medical cannabis. Regarding possible negative effects, a lower score indicates greater concern. Items about legalization were dichotomous, whereby 1=yes and 2=no). Range b: Indicates the Likert-type scale score range for each item of the scale.

Scale	n	Mean (SD)^a^	Range^b^
Perceived Knowledge of Medical Cannabis Scale (PK-S)	526	3.17 (1.17)	1-6
Concern About Possible Negative Effects of Medical Cannabis Scale (CNE-S)	526	3.39 (1.10)	1-6
Perceptions About Medical Cannabis Use Scale (PU-S)	526	2.10 (.797)	1-6
Should medical marijuana be legal in all 50 states in the United States?	460	1.05	1-2
Should recreational marijuana be legal in the United States?	440	1.22	1-2

*Opinions About Legalization* 

A substantial proportion of participants reported that MC should be nationally legalized (n=439; 90.5%), and fairly less than those (n=343; 70.7%) reported recreational cannabis should be nationally legalized.

Correlations and regression model

Table 3 reports the results of the point-biserial correlation analysis for preliminary hypothesis testing. There were correlations between the study variables perception of knowledge about MC, concern for possible negative effects of MC use, perceptions about MC, belief in federal legalization of MC, and belief in the federal legalization of recreational cannabis; all correlations were statistically significant, p < .05.

**Table 3:** Point-biserial Correlations between major study variables ** Correlation is significant at the 0.01 level (2-tailed) * Correlation is significant at the 0.05 level (2-tailed)

	Perceived Knowledge	Concern for Negative Effects	Perceptions	Believe MC should be legal in all 50 states in the U.S	Believe recreational marijuana should be legal in the U.S.
Perceived Knowledge	--				
Concern for Possible Negative Effects	-.378**	--			
Perceptions	.506**	-.324**	--		
Believe MC should be legal in all 50 states in the U.S.	.095*	-.278**	.412**	--	
Believe recreational marijuana should be legal in the U.S.	.369**	-.488**	.538**	.359**	--

Table 4 reflects the findings from a multiple linear regression that was calculated to predict medical students’ perceptions of MC. A significant regression equation was found: (F(4, 428) = 114.826, p < .001 with an R2 = 0.518 [adjusted R2 = 0.513]). Greater perception of knowledge about MC, lower concern for possible negative effects of MC use, greater belief in federal legalization of MC, and greater belief in the federal legalization of recreational cannabis were associated with more positive perceptions about MC. The percentage of variance in the scores accounted for by the model was 51%. The combination of independent variables representing modifiable behaviors (i.e., knowledge, concerns) predicting even a moderate amount of the variance in a relevant outcome could be significant.

**Table 4:** Linear regression model predicting medical students’ perceptions about medical cannabis Dependent variable: Perceptions about medical cannabis

	B	SE	β	t	P-value
(Constant)	0.523	0.228		2.300	.022
Perceived Knowledge of Medical Cannabis	0.227	0.026	0.333	8.846	< .001>
Concern about Possible Negative Effects of Medical Cannabis	-0.175	0.029	-0.242	-5.962	< .001>
Belief in U.S. Federal Legalization of Medical Cannabis	0.912	0.131	0.254	6.964	< .001>
Belief in U.S. Federal Legalization of Recreational Cannabis	0.377	0.076	0.199	4.939	< .001>

## Discussion

Overview of the major findings

This study provided a multivariate analysis predicting medical students’ perceptions of MC. It was found that higher levels of perceived knowledge, less concern for possible adverse effects, and greater belief in federal legalization for both recreational and medical use of cannabis predicted higher levels of positive perceptions of MC in this sample of medical students. Participants had only moderate knowledge about and familiarity with the utility of MC. They also reported concern about the potential of MC use leading to addiction. More notably, it was also found that medical students supported legalization of both recreational and medical cannabis, although in different proportions. The mean score for the PU-S was 2.10 (on a scale of 1 to 6, with higher scores indicating more positive attitudes toward MC), which was considerably low, indicating the participants had, overall, a less positive outlook of MC. Questions in the PU-S had more to do with what a physician can/should do and beliefs about the benefits of using MC, which are highly subjective than undetectable social, cultural, and religious/spiritual influences; these items are dissimilar from more objective questions such as legalization, general knowledge about MC, and detrimental effects of use. Moreover, messages students may have received from professors and mentors at school may have influenced these findings, as physicians may have less favorable perceptions about MC than medical students.

Perceived Knowledge of MC

Greater level of perceived knowledge was a statistically significant contributor to positive perceptions about MC. Participants felt, overall, they had moderate substantial knowledge of MC, felt ‘somewhat confident” regarding their current knowledge of MC, and reported they believed MC was effective for treating certain medical conditions, primarily chronic pain, cancer, and terminal illness. It was not surprising that higher levels of knowledge about MC would be associated with more positive perceptions about it, particularly in medical students who are just learning to accurately assess pain and treat chronic pain as an essential skill. Moreover, empathy for the ill may endear them to be more acutely aware of others’ pain and discomfort.

Concern for Possible Negative Effects

Participants in this sample reported a moderate amount of concern for the possible negative effects of MC use, including abuse/misuse and its potential for addiction. The regression model indicated more concern for potential adverse effects of MC use was a statistically significant contributor to non-favorable perceptions toward MC. This finding was not surprising given the United States Drug Enforcement Administration has classified cannabis as a drug that has no accepted medical use but holds potential for abuse. Other substances that are also currently classified as a Schedule I drug include lysergic acid diethylamide (LSD), heroin, and 3,4-methylenedioxymethamphetamine (ecstasy). The importance of training medical students to effectively assess and treat substance use disorders has become increasingly recognized. However, it was expected that medical students, who are generally much younger and thus perhaps more open-minded and informed about cannabis, would have fewer concerns about its use, considering the perceived harmfulness of cannabis has decreased significantly in the past three decades [24]. More research is needed to explore the nuances regarding concerns among medical students regarding the potential hazards of MC while the general public increasingly sees cannabis as harmless [21].

Opinions About Legalization

Greater favor for the legalization of both medical and recreational cannabis was a statistically significant contributor to positive perceptions about MC. The survey contained items related to opinions about the federal legalization of both MC and recreational cannabis. As expected, due to the nature of the sample (e.g., young adults) the majority of participants thought cannabis should be nationally legalized. These findings are supported by previous research indicating younger adults may lean towards more progressive attitudes toward cannabis regulation [23]. Interestingly, this study found more students supported the legalization of cannabis for medical purposes over recreational ones (90.5% vs. 70.7%, respectively), which may be due to the fact that in Florida where this sample was drawn from, MC is decriminalized whereas recreational cannabis is not.

Limitations

First, cross-sectional survey designs cannot predict changes over time or cause-and-effect relationships and limit the ability to generalize findings to all medical students. Second, data collection from both osteopathic and allopathic schools of medicine and/or multi-site data collection might have yielded different results. It is possible that medical students from other schools than the one used for this study will have different opinions that are not represented in this sample. Last, conducting research using electronic questionnaires can hamper participant availability or willingness to answer questions without the researcher there.

## Conclusions

In this study, medical students with higher levels of perceived knowledge of MC, less concern for its potential negative effects, and greater belief in federal legalization of cannabis for both medical and recreational purposes were associated with more positive perceptions about MC. Participants reported moderate knowledge and familiarity with MC and its efficacy for health conditions and reported concern about the potential for misuse and/or addiction. Although the majority of students had positive perceptions about cannabis legalization, they were more apt to endorse it for medicinal use over recreational use. Changes regarding the legalization of MC across the United States will most likely continue and controversies over its efficacy will ensue. Even in states where MC is illegal, the development of curricula and training programs are encouraged as many students enter post-graduate training programs in states other than those for their undergraduate training. As the interest in the therapeutic effects of cannibals increases, it is important to establish its efficacy for medicinal use and proper dosing, and evaluate its potential for adverse effects so physicians in training can be equipped with the best available evidence regarding treatment decisions. Nonetheless, the findings from this study may help guide medical educators to enhance students’ readiness to provide MC as a viable treatment when indicated.
